# Ectopic Expression of *VvSUC27* Induces Stenospermocarpy and Sugar Accumulation in Tomato Fruits

**DOI:** 10.3389/fpls.2021.759047

**Published:** 2021-11-17

**Authors:** Yumeng Cai, Ling Yin, Wenrui Tu, Zhefang Deng, Jing Yan, Wenjie Dong, Han Gao, Jinxu Xu, Nan Zhang, Jie Wang, Lei Zhu, Qingyong Meng, Yali Zhang

**Affiliations:** ^1^College of Food Science and Nutritional Engineering, China Agricultural University, Beijing, China; ^2^Tianjin Key Laboratory of Crop Genetics and Breeding, Crops Research Institute, Tianjin Academy of Agricultural Sciences, Tianjin, China; ^3^Guangxi Crop Genetic Improvement and Biotechnology Key Laboratory, Academy of Agricultural Sciences, Nanning, China; ^4^College of Food Science and Technology, Heilongjiang Bayi Agricultural University, Daqing, China; ^5^The State Key Laboratory for Agrobiotechnology, College of Biological Sciences, China Agricultural University, Beijing, China

**Keywords:** *Vitis vinifera*, *VvSUC*, overexpression, tomato, stenospermocarpy, transcriptomic analysis

## Abstract

Seedless fruits are favorable in the market because of their ease of manipulation. Sucrose transporters (SUTs or SUCs) are essential for carbohydrate metabolism in plants. Whether SUTs participate directly in causing stenospermocarpy, thereby increasing fruit quality, remains unclear. Three *SUTs*, namely, *VvSUC11*, *VvSUC12*, and *VvSUC27* from *Vitis vinifera*, were characterized and ectopic expression in tomatoes. *VvSUC11*- and *VvSUC12*-overexpressing lines had similar flower and fruit phenotypes compared with those of the wild type. *VvSUC27*-overexpressing lines produced longer petals and pistils, an abnormal stigma, much less and shrunken pollen, and firmer seedless fruits. Moreover, produced fruits from all *VvSUC*-overexpressing lines had a higher soluble solid content and sugar concentration. Transcriptomic analysis revealed more genes associated with carbohydrate metabolism and sugar transport and showed downregulation of auxin- and ethylene-related signaling pathways during early fruit development in *VvSUC27*-overexpressing lines relative to that of the wild type. Our findings demonstrated that stenospermocarpy can be induced by overexpression of *VvSUC27* through a consequential reduction in nutrient delivery to pollen at anthesis, with a subsequent downregulation of the genes involved in carbohydrate metabolism and hormone signaling. These commercially desirable results provide a new strategy for bioengineering stenospermocarpy in tomatoes and in other fruit plants.

## Introduction

Carbon fixation occurs in photosynthetic source organs, and the products are exported as sucrose to non-photosynthetically heterotrophic sink organs (Koch, 2004). Sucrose is essential for plant growth and is a primary driver of crop yield. Therefore, the use of SUTs in the translocation of sucrose is required for plant development. Many SUTs have been identified and characterized in both monocots and dicots, playing a role in the loading of sucrose into the phloem and sink tissues as well as the accumulation of soluble sugars in cells (Reinders et al., 2006).

Sugar is a key component in the sweet taste of fruits and can improve their flavor. Three *SUTs* genes have been isolated in tomatoes (*Solanum lycopersicum*, previously *Lycopersicon esculentum*), which have been reported to be localized in sieve elements (SE): *SlSUT1*, *SlSUT2*, and *SlSUT4* (Barker et al., 2000; Weise et al., 2000). SlSUT4 is expressed predominantly in female flowers and green tomato fruits; however, SlSUT1 and SlSUT2 accumulate in the SEs of young and mature tomato fruits (Weise et al., 2000; Hackel et al., 2006). The antisense inhibition of both *SlSUT1* and *SlSUT2* prevents the production of normal tomato fruits. The phloem loading and supply to terminal sink organs of *SlSUT1* antisense plants is disturbed and delays the development of storage sink organs, whereas *SlSUT2* antisense inhibition could affect tomato fruit (Hackel et al., 2006). Furthermore, *CitSUT1* and *CitSUT2* have been isolated from sweet oranges (*Citrus sinensis*). *CitSUT1* is strongly expressed in the source and sugar-exporting organs. In contrast, *CitSUT2* is expressed more strongly in the sink and sugar-importing organs (Yao Li et al., 2003). Additionally, melon SUT1 catalyzes the active loading of sucrose into the phloem (Gil et al., 2011). Cucumber (*Cucumis sativus*) *CsSUT1*-RNA interference (RNAi) lines exhibited a decreased content of sucrose, hexose, and starch with male sterility (Sun et al., 2019), whereas suppressing *CsSUT2* decreased the sucrose level and increased the stachyose content in leaves (Ma et al., 2019b).

Sugar efflux transporters are essential for the maintenance of plant nectar, seed, and pollen development (Huang et al., 2010). The modified subcellular sugar compartmentation, altered cellular sugar sensing, and affected assimilate allocation could affect the biomass of seeds (Wingenter et al., 2010). Therefore, sugar transport might influence seed abundance. It has been reported that the expression of *VfSUTl* from fava beans (*Vicia faba*) is specific to outer epidermal cells and could be induced by carbohydrate availability during epidermal contact of the growing embryo with the seed coat (Weber et al., 1997). SUT1 has been reported to be essential for loading sucrose into the phloem *via* an apoplastic route, as well as for intermesophyll transport. In tobacco plants transformed by the *SUT1* antisense construct, the most strongly affected plants and more pronounced phenotypes (curl leaves and chlorotic or even necrotic rims and intercostal fields) could not produce seeds, suggesting that the antisense plants were unable to export their stored sugar reserves (Burkle et al., 1998). Furthermore, SlSUT2 could be directly involved in phloem unloading at the level of tomato fruits or seeds; *SUT2* antisense inhibition could therefore disturb pollination to produce seedless tomato fruit (Hackel et al., 2006). However, there are no reports on the effect of *SUT* overexpression on seed formation.

Overexpression of *SUT* genes provides genetic evidence of their essential roles in fruit sugar accumulation. The *MdSUT2.2*-overexpressing lines (apple, *Malus domestica*, formerly MdSUT2) accumulated more total soluble sugars and sucrose and grew faster than the wild-type control (Ma et al., 2017, 2019a). The ectopic expression of *PbSUT2* (*Pyrus bretschneideri*) in tomato plants could lead to early flowering of transgenic tomatoes, higher fruit quantity, lower plant height, increased net photosynthetic rate in leaves, and higher sucrose content in mature fruit, whereas it decreases the contents of glucose, fructose, and total soluble sugars in mature fruits (Wang et al., 2016).

*Vitis* has various vining species, which are used worldwide as medicinal herbs and in winemaking (Chen et al., 2018). The organoleptic quality of the berries, flavor, and stability of wine is determined by the type and concentration of sugars and acids in grapes (Chen et al., 2015). In grape berries, three functional SUTs, namely, VvSUC11, VvSUC12, and VvSUC27, have been identified (Davies et al., 1999). During berry development, the weakest expression of *VvSUC11* and *VvSUC12* was observed at the stage of fruit setting, where similar expression levels were shown. In contrast, *VvSUC27* showed the highest expression level at the fruit set stage (Afoufa-Bastien et al., 2010). In our previous study, we concluded that the expression of plasma membrane-located VvSUC27 was negatively correlated with sugar accumulation in grape berries (Cai et al., 2017). The influence of *VvSUC11*, *VvSUC12*, and *VvSUC27* on plant growth has been analyzed in our previous studies (Cai et al., 2017, 2019, 2020). However, the influence of each *VvSUC* on fruit quality is poorly understood. Considering the inefficient transformation and the relatively long growth cycle of grape, we chose Micro-Tom instead. Micro-Tom is a miniature dwarf tomato, which is suitable for the analysis of fruit morphology; it can grow at high densities, be transformed efficiently, and has a short life cycle (Martinelli et al., 2009). Here, we obtained three types of *VvSUC*-overexpressing tomatoes (Micro-Tom). Overall, the three types of *VvSUC*-overexpressing tomatoes significantly improved the sugar content of fruits. Ectopic expression of *VvSUC27* in tomatoes produced larger flowers with shrunken pollen and transgenic seedless fruits with improved fruit firmness. In addition, the ectopic expression of *VvSUC27* reduced the expression of auxin- and ethylene-related synthesis or responsiveness genes of fruits. These findings support the hypothesis that *VvSUC27* plays a central role in fruit seed development and provides desirable traits in commercially grown fruit crops.

## Materials and Methods

### Plant Material and Growth Conditions

The leaves from the *V. vinifera* “Thompson Seedless” variety were collected in Shangzhuang (Beijing, China). The leaves were harvested, immediately frozen in liquid nitrogen, and stored at −80°C.

The tomato plant materials used in this study were of the Micro-Tom background. Tomato cells were cultured in a growth chamber with 16-h light at 20–25°C.

### RNA Extraction and qRT-PCR

Total RNA from grape berry and transgenic tomato tissues was isolated using HiPure Plant RNA Kits (Magen, China). Then, cDNA was synthesized using the HiFiScript gDNA Removal cDNA Synthesis Kit (CWBIO, China). To normalize the cDNA samples, *SlEF-1α*, *SlGADPH*, and *SlActin* from tomatoes were selected as internal controls. Gene-specific primers (Supplementary Table S1) were designed, and qRT-PCR was performed using an UltraSYBR Mixture Kit (CWBIO, China) with a Rotor-Gene^®^ SYBR^®^ Green PCR Kit (QIAGEN, Germany). To determine the different concentrations of cDNA, the threshold cycle for each qPCR was identified and compared against the internal standards (*SlEF-1α*, *SlGADPH*, and *SlActin* for tomatoes).

### Generation of Transgenic Plasmid Constructs and Plant Transformation

To generate the 35S:: *VvSUC11*, *VvSUC12*, and *VvSUC27* constructs, the full-length coding sequences of *VvSUC11*, *VvSUC12*, and *VvSUC27* were amplified from *V. vinifera* “Thompson Seedless” cDNA using the Gateway-PCR specific primers (added attB1 and attB2, underlined in Supplementary Table S1), and the overexpression *SUT* constructs were generated by Gateway-PCR (Invitrogen). The resulting vectors [pH7WG2D, 1- *VvSUC11*; pH7WG2D,1- *VvSUC12*; and pH7WG2D,1- *VvSUC27* constructs (35S promoter)] were obtained and confirmed by sequencing for plant transformation.

The final verified constructs were introduced into transgenic Micro-Tom tomatoes using the *Agrobacterium*-mediated transformation method, as previously described (Van Eck et al., 2006). A hygromycin selection marker was used to identify transgenic tomato lines. T_1_ lines of *VvSUC11*- and *VvSUC12*-OE lines and T_0_ lines of *VvSUC27*-OE lines were used for further phenotypic analyses.

### Scanning Electron Microscopy

Tomato pollen grains were isolated and attached to double-sided adhesive tape (Plano), which was set on SEM aluminum specimen holders. Then, the specimens were coated with gold for 60s. An FEI Quanta 200 scanning electron microscope was used for SEM.

### Histological Analyses With Toluidine Blue Staining

Crosswise cutting of seeds as well as anthesis, 5, and 9days post-anthesis (DPA) fruit sections were set on glass slides and analyzed with toluidine blue, as previously described (Greco et al., 2012). The cells were observed under an Axio Scope A1 microscope.

### Determination of the Number of Pollen Grains Under the Microscope

The number of pollen grains from the flower at anthesis was measured under a microscope. First, the freshly bloomed flower was removed, chopped on a clean plate with a sterile surgical blade, and placed in a 2-mL centrifuge tube. The plate was then washed with a small amount of ddH_2_O. The washed ddH_2_O was transferred to a centrifuge tube, and water was added to a total of 100μl. After vortexing for 5min, the suspension was briefly centrifuged and homogenized by pipetting. Then, 100μl of the suspension was sucked into the counting area of a clean blood cell counting plate using a pipette, and the coverslip was added to avoid air bubble generation. The blood cell counting plate was placed on the stage of the microscope and clamped securely until the pollen was no longer suspended in the liquid. After finding the counting area under the low-power lens, it was switched to a high-power lens to observe and count the pollen. The number of pollen cells in each flower was calculated using the formula: N/25×5×10×100.

### Texture Measurement

Firmness characteristics were measured for 20 intact tomato fruits from each line and harvested at the mature stage. The measurements were performed as previously described with some modifications (Smith et al., 2002). Force-deformation was recorded using a TMS-PRO Texture Analyzer (FTC, United States). Firmness was defined as the force at maximum deformation (2mm). The probe diameter was 2mm, the test speed was 60mm/min, the return speed was 120mm/min, and the test depth was 2mm. The starting force was 0.5N (Newton). The firmness was measured at an equal distance at three points at the equator of each fruit. The average value was then determined.

### Soluble Solid Content Measurements

Fruit sap from different growth phases of tomato fruits was collected using a garlic press, and the refractive index (Brix) was determined with a refractometer (ATAGO).

### Titratable Acidity Measurements

The fruit was collected at the mature fruit stage. The samples were prepared as follows. 5g of fruit was ground and mixed in distilled water and then fixed capacity to 250ml. 50ml filter fluid was picked and titrated with 0.1mol/l NaOH solution to pH 8.1 by using phenol as an indicator. Titratable acidity was calculated according to the consumption of standard alkali liquid. The total soluble solid/titratable acidity ratio was determined by dividing % the total soluble solid by % titratable acidity.

### Analysis of Sugars in Tomato Fruits

The fruit was collected at the mature fruit stage. Sugar extraction and concentration measurements were performed by high-performance liquid chromatography (HPLC), as previously described with some modifications (Agius et al., 2018). First, 0.5/1.0g of fruit was ground with 80% ethanol, and then placed in an 80°C water bath for 1h, and centrifuged at 8,000×g for 10min, and the supernatant was collected. Then, 80% ethanol was continually added to the precipitate; this step was repeated twice in an 80°C water bath for 30min. The supernatants were combined several times and were then evaporated and dissolved in 2ml of distilled water, shaken to mix, and centrifuged at 13,000×g for 2min. The resulting mixture was then filtered through a 0.22μm microporous membrane and used for HPLC.

Agilent 1260 (Agilent 1260 series) HPLC was used to determine the sugar content. A sugar analysis column (Aminex HPX-87H column, Bio-Rad) and a refractive index detector were used with the 6mm H_2_SO_4_ mobile phase at a flow rate of 0.5ml/min and a column temperature of 35°C.

### RNA-Seq Analysis

The quality of clean reads obtained by RNA-seq was checked to ensure that they were credible for a series of analyses. Subsequently, all clean reads were mapped to the tomato reference genomes using TopHat2.^1^ Fragments per kilobase of transcript per million fragments mapped (FPKM) was used to show the expression level value. The fold change was calculated using the FPKM VvSUC-OE line/FPKM WT. The selection criteria were |fold change|>2 and false discovery rate (FDR)<0.01. The clean data for this paper have been submitted to the National Center for Biotechnology Information Sequence Read Archive under accession number GSE161505.^2^

### Statistical Analyses

One-way ANOVA and Tukey’s test were performed to analyze the significance of data using SPSS v16.0 (SPSS Corp., IL, United States). Statistical significance was set at *p*<0.05.

## Results

### Ectopic Expression of *VvSUC27* Induced Larger Flowers and Altered the Phenotype of the Style and Pollen

**Figure 1:**
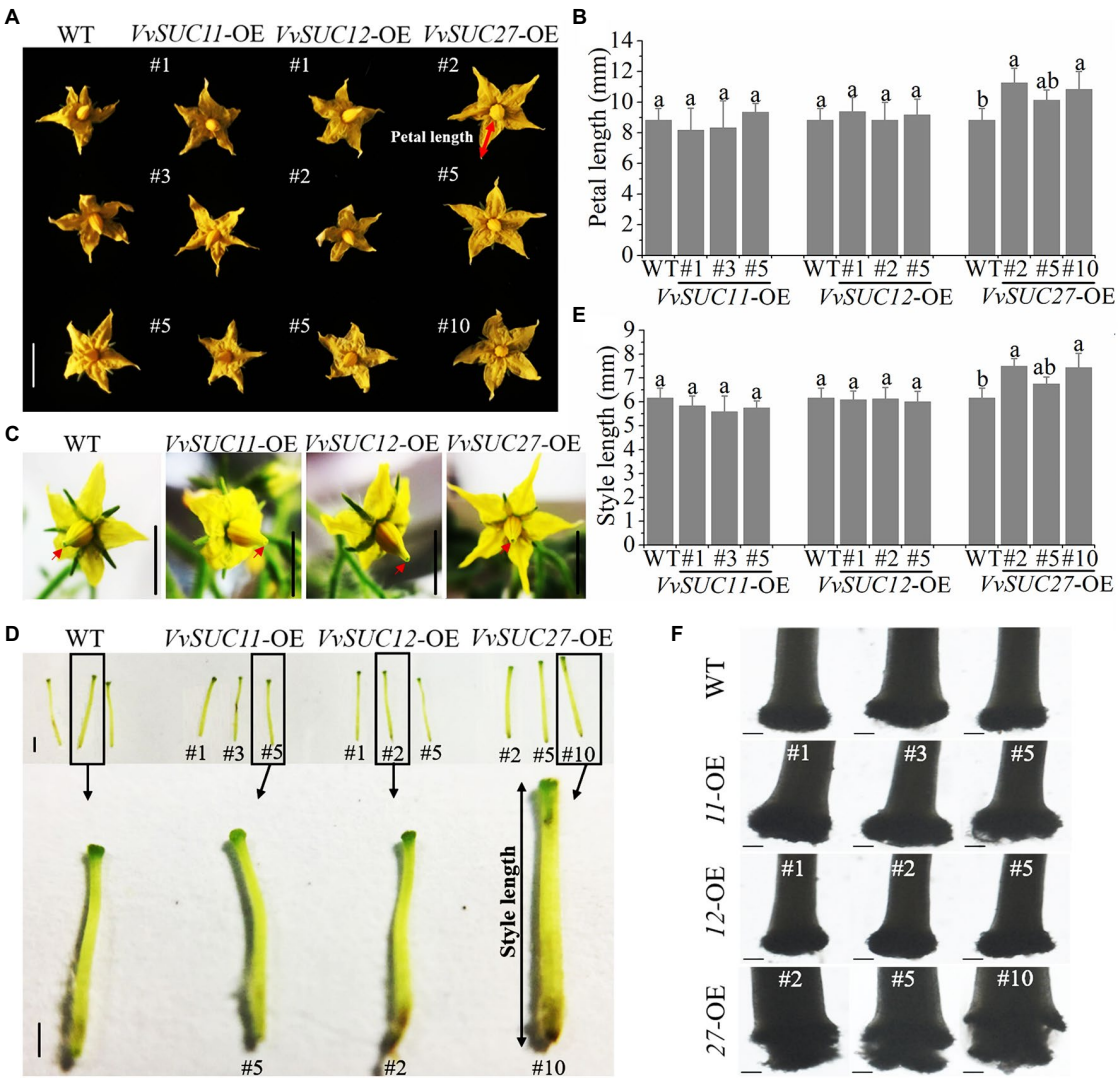
Phenotype of *VvSUC*-OE tomato flowers. **(A)** Comparisons between WT and each *VvSUC*-OE line regarding the phenotype of flowers. Scale bar, 1cm. **(B)** Comparisons between WT and each *VvSUC*-OE line for petal length. Petal length was measured using the red arrow shown in **(A)**. Error bars indicate mean±SD of ten biological replicates. Different lowercase letters show statistically significant differences between WT and each *VvSUC*-OE line (Tukey’s test, *p*<0.05). **(C)** Close observation of the flowers of the WT and each *VvSUC*-OE line. Scale bar, 1cm. The stigmas are indicated by red arrows. **(D)** Comparisons between the WT and each *VvSUC*-OE line regarding style phenotype. Scale bar, 100μm. **(E)** Comparison between the WT and each *VvSUC*-OE line regarding the style length. Style length was measured using the black arrow shown in **(D)**. Error bars indicate mean±SD of ten biological replicates. Different lowercase letters show statistically significant differences between the WT and each *VvSUC*-OE line (Tukey’s test, *p*<0.05). **(F)** Observation of the stigma of the WT and *VvSUC*-OE lines by microscopy. Scale bar, 100μm.

*VvSUC11*, *VvSUC12*, and *VvSUC27* from *Vitis vinifera* were chosen for investigation using *Solanum lycopersicum “*Micro-Tom” because they have different gene expression patterns and properties (Manning et al., 2001; Zhang et al., 2008; Afoufa-Bastien et al., 2010; Cai et al., 2019) and all have a close correlation with sugar accumulation in the fruit. T_0_ further characterize the biological function of *VvSUC11*, *VvSUC12*, and *VvSUC27* in fruit berries, the overexpression vectors 35S:: *VvSUC11*, *VvSUC12*, and *VvSUC27* were constructed and transformed into *Solanum lycopersicum* cv. Micro-Tom. Then, the T_0_ transgenic lines of each *SUT*-overexpressing (OE) tomato were obtained. In total, 13 *VvSUC11*-OE transgenic tomato lines, 15 *VvSUC12*-OE transgenic tomato lines, and 18 *VvSUC27*-OE transgenic tomato lines were produced and determined by PCR analysis of genomic DNA. Among them, *VvSUC11*- and *VvSUC12*-OE tomatoes could produce normal seeds, whereas *VvSUC27*-OE tomatoes were seedless (Supplementary Figure S1). Therefore, three lines of each T_1_ transgenic *VvSUC11*- and *VvSUC12*-OE lines and T_0_ transgenic *VvSUC27*-OE lines were randomly chosen for further investigation, verified by PCR (Supplementary Figure S2A), and further confirmed by RT-qPCR (Supplementary Figure S2B). These lines were subsequently used for further investigation. On the whole, most of phenotypes from *VvSUC27*-OE lines were different from *VvSUC11*- and *VvSUC12*-OE lines. It is worth noting that the relative expression of both *VvSUC11* from *VvSUC11*-OE lines and *VvSUC12* from *VvSUC12*-OE lines was higher than that of *VvSUC27* from *VvSUC27*-OE lines (Supplementary Figure S2B). Therefore, it could be inferred that unique phenotypes from *VvSUC27*-OE transgenic plants could be due to their different protein structure and enzyme activity rather than just high expression level in these transgenic lines. In terms of the phenotype of transgenic tomato flowers, *VvSUC11*- and *VvSUC12*-OE lines had similar flower phenotypes, whereas *VvSUC27*-OE lines presented a promoted phenotype compared with that of the wild type (WT; Figure 1). The petal length of the *VvSUC27*-OE lines was significantly longer than that of the WT (Figures 1A,B). Close observation of the flowers of the WT and *VvSUC*-OE lines showed that only the *VvSUC27*-OE stigma was wrapped in stamens, and stigma could not be exposed out from stamen (Figure 1C). Furthermore, flowers were emasculated and pistils were isolated from flowers (Figure 1D), and the lengths of *VvSUC27*-OE pistils were much longer than those of *VvSUC11*- and *VvSUC12*-OE pistils, as well as that of the WT (Figure 1E). Next, the stigmas were observed under an Axio Scope A1 (ZEISS) microscope, and only *VvSUC27*-OE stigmas showed abnormality for unconsolidated shape (Figure 1F).

**Figure 2:**
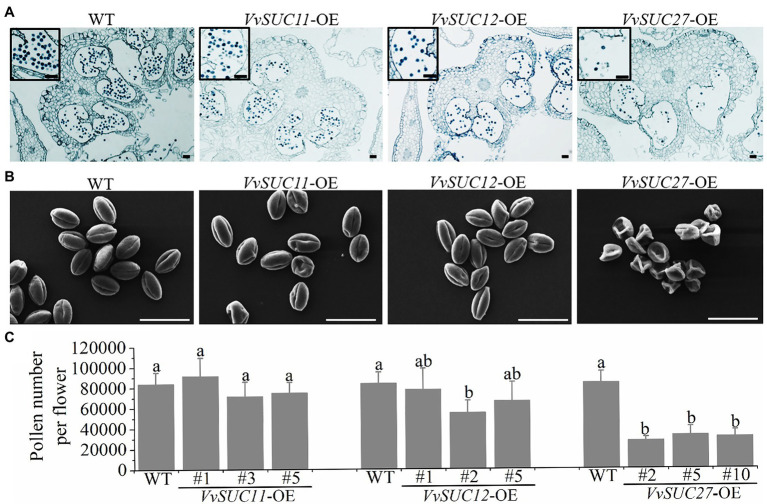
Phenotype and number of *VvSUC*-OE tomato pollen grains. **(A)** Observation of the corollas of the WT and *VvSUC*-OE lines by microscopy. The pollen grains were enlarged and marked with black boxes. Scale bar, 100μm. **(B)** Observation of the pollen of the WT and *VvSUC*-OE lines by scanning electron microscopy (2,000×). Scale bar, 50μm. **(C)** Comparison between the WT and each *VvSUC*-OE line on the number of pollen grains per flower. Error bars indicate mean±SD of six biological replicates. Different lowercase letters show statistically significant differences between the WT and each *VvSUC*-OE line (Tukey’s test, *p*<0.05).

The corollas of the WT and *VvSUC*-OE lines were stained with 0.5% toluidine blue solution and observed under a microscope (Figure 2A). Compared with the WT, the pollen of *VvSUC11*- and *VvSUC12*-OE lines were normal, whereas that of *VvSUC27*-OE lines was less numerous and abnormal. Scanning electron microscopy (SEM) was used to further observe the pollen morphology of the WT and each *VvSUC*-OE line (Figure 2B). No significant differences were observed between the WT and *VvSUC11*- or *VvSUC12*-OE pollen morphology. Most pollen was oval and full, with regular shapes. However, all the pollen grains of the *VvSUC27*-OE lines were shrunken. The sole flowers of the WT and each *VvSUC*-OE line were cut, dissolved, and shaken using a vortex mixer. The pollen grains for each flower were counted using a hemocytometer under a microscope (Figure 2C). Regarding pollen number, there was no significant difference between the WT and *VvSUC11*-OE lines. Among *VvSUC12*-OE lines, only that of *VvSUC12*-OE-2 pollen grains was lower than that of the WT. However, all *VvSUC27*-OE lines were significantly reduced compared with WT. The results of *in vitro* tests showed that over 90% of the WT pollens could germinate normally, while only 15% could in *VvSUC27*-OE lines (Supplementary Figure S3).

### Ectopic Expression of *VvSUC27* Dramatically Improved Fruit Firmness

**Figure 3:**
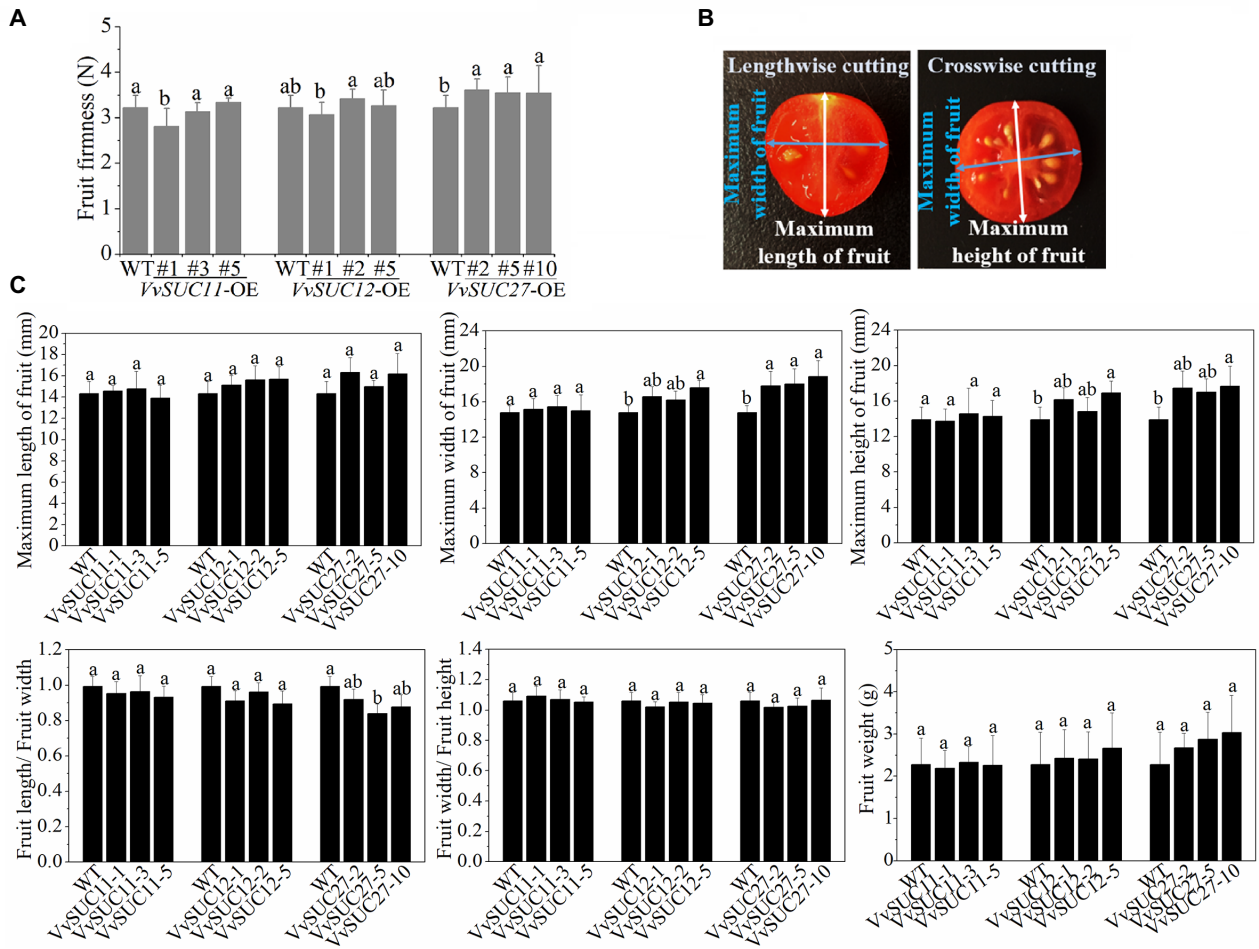
Phenotype, firmness, and weight of *VvSUC*-OE tomato fruits. **(A)** Comparison between the WT and each *VvSUC*-OE line regarding fruit firmness. **(B)** Diagram of the maximum length, width, and height of fruits. **(C)** The maximum length, width, height, length/width ratio, width/length ratio, and fruit weight of WT and *VvSUC*-OE tomato fruit. Error bars indicate the mean±SD of twenty biological replicates. Different lowercase letters show statistically significant differences between the WT and each *VvSUC*-OE line (Tukey’s test, *p*<0.05).

In the present study, the growth phase of the WT and each *VvSUC*-OE line was measured. However, consistent with those of the WT, fruits of each *VvSUC*-OE line all ripened normally at nearly 54 DPA and exhibited similar phenotypes. Fruit firmness was determined in this study. The results suggested that fruit firmness decreased only in the *VvSUC11*-OE-1 line, whereas other transgenic lines from the *VvSUC11*-OE and *VvSUC12*-OE lines remained unchanged compared with that of the WT (Figure 3A). However, the fruit firmness of all *VvSUC27*-OE lines was significantly increased compared with the WT: by 1.12-, 1.10-, and 1.10-fold for *VvSUC27*-OE-2, 5, and 10, respectively (Figure 3A). The length, width, and height of the fruits were measured, as shown in Figure 3B. The length of *VvSUC*-OE fruits showed no significant changes (Figure 3C). The width and height of all the *VvSUC11*-OE and *VvSUC12*-OE lines (except for *VvSUC12*-OE-5) were also similar to those of the WT. However, the width was highly increased in the *VvSUC27*-OE lines, and the height was significantly increased in *VvSUC27*-OE-10, which resulted in a decreased length/width ratio in *VvSUC27*-OE-5. This was consistent with the width/height ratio, indicating that the fruit shape remained unchanged (Figure 3C). No significantly greater weight of *VvSUC27*-OE fruits was observed compared with WT fruits (Figure 3C).

### Ectopic Expression of All Three *VvSUCs* Significantly Increased Fruit Sugar Concentration

**Figure 4:**
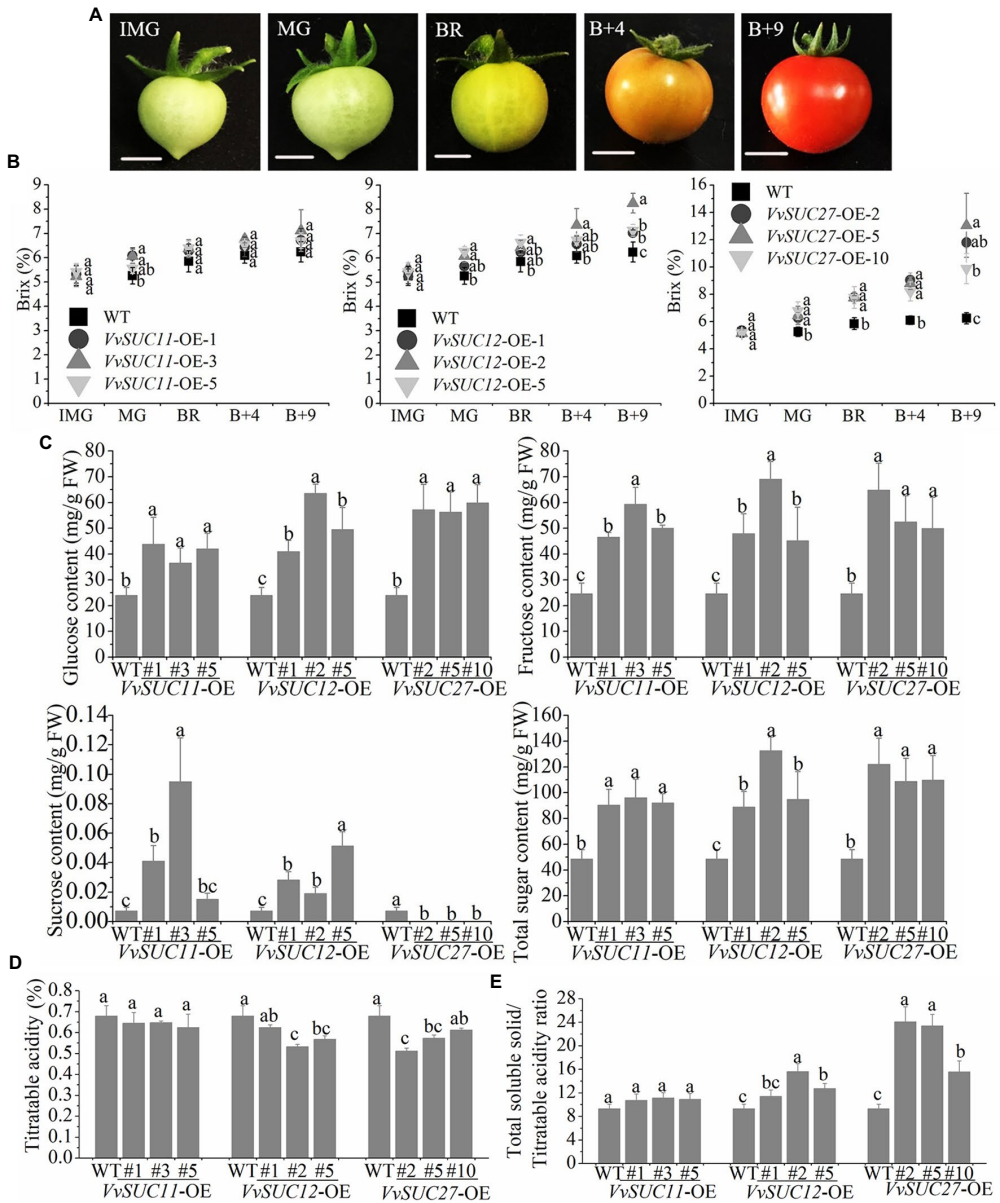
Sugar concentration of the *VvSUC*-OE tomato fruit. **(A)** Fruit development in tomatoes. IM, immature; MG, mature green; BR, breaker; B+4: 4 d after fruit color break; B+9: 9 d after fruit color break. Scale bar, 50mm. **(B)** Comparison between the WT and each *VvSUC*-OE line regarding different tomato growth phases of soluble solid content. **(C)** Comparison between the WT and each *VvSUC*-OE line regarding glucose, fructose, and total sugar concentrations in tomato fruits at the red ripe stage. FW, Fresh weight. **(D)** Comparison between the WT and each *VvSUC*-OE line regarding titratable acidity. **(E)** Comparison between the WT and each *VvSUC*-OE line regarding the total soluble solid/titratable acidity ratio. Error bars indicate the mean±SD of nine biological replicates. Different lowercase letters show statistically significant differences between the WT and each *VvSUC*-OE line (Tukey’s test, *p*<0.05).

SUTs play key roles in the translocation of sucrose both in phloem loading from the source tissue and sucrose unloading into sink tissue (Rennie and Turgeon, 2009). However, how VvSUC affects fruit sugar accumulation remains unknown. Here, the soluble solid content of tomatoes from different growth phases (Figure 4A) was measured using a refractometer in Brix units (Figure 4B). In total, the Brix values of the *VvSUC*-OE fruits were all similar to those of WT fruits during the immature period. The Brix of *VvSUC11*-OE fruits was nearly consistent with that of WT fruits during the growth phase. Only the Brix of *VvSUC12*-OE-2 was higher than that of the WT across the growth phase. The final Brix of fruits for the *VvSUC12*-OE-1, 2, and 5 lines increased by 1.13-, 1.32-, and 1.14- fold that of the WT, respectively. For *VvSUC27*-OE fruits, the Brix of all lines was significantly improved compared with that of the WT across the growth phase. The final Brix of fruit 9 d after the color break for the *VvSUC27*-OE-2, 5, and 10 lines and the WT was 11.80, 13.05, 9.89, and 6.24%, respectively. These represent increases of 1.89-, 2.09-, and 1.58-fold that of the WT, respectively.

Sugar analyses of *VvSUC*-OE tomato fruits at the red ripe stage were performed by HPLC. HPLC confirmed the obvious improvement in glucose, fructose, sucrose, and total sugar (composed of glucose, fructose, and sucrose) concentrations in all tested *VvSUC*-OE lines (Figure 4C). Glucose and fructose are the main soluble sugars in tomato fruits after ripening. Among the *VvSUC*-OE lines, *VvSUC11*-OE fruits had the lowest glucose content. For the *VvSUC11*-OE-1, 3, and 5 lines, the glucose concentration of fruits was 1.83-, 1.52-, and 1.75-fold higher than that of the WT, respectively. The glucose content of the *VvSUC27*-OE-2, 5, and 10 lines was 2.38, 2.34, and 2.49-fold higher than that of the WT, respectively. The glucose content of the *VvSUC12*-OE lines (*VvSUC12*-OE-1, 2, and 5 fruits) was between that of the *VvSUC11*-OE lines and *VvSUC27*-OE lines, which were 1.71-, 2.65-, and 2.06-fold higher than that of WT, respectively. In terms of fructose content in tomato fruits, that of all *VvSUC*-OE lines was significantly increased. For the *VvSUC11*-OE-1, 3, and 5 lines, the fructose content of tomato fruits was 1.89-, 2.4-, and 2.03-fold higher than that of the WT, respectively. For the *VvSUC12*-OE lines, the fructose contents of the *VvSUC12*-OE-1, 2, and five fruits were 1.95-, 2.80-, and 1.95-fold higher than that of the WT, respectively. Similarly, the fructose content of the *VvSUC27*-OE-2, 5, and 10 fruits was 2.63-, 2.13-, and 2.03-fold higher than that of the WT, respectively. Mature tomato fruits contain only trace amounts of sucrose. The sucrose content of tomato fruits in *VvSUC11*-OE-1, 3, and 5 lines (0.041, 0.095, and 0.015mg/g) was significantly higher than that of the WT (0.0073mg/g). For the *VvSUC12*-OE lines, the sucrose content of tomato fruits in the *VvSUC12*-OE-1, 2, and 5 fruits was 0.028, 0.019, and 0.051mg/g, respectively. In contrast, no sucrose was detected in the *VvSUC27*-OE fruits. The total sugar content was calculated using glucose, fructose, and sucrose. Overall, the total sugar content of all *VvSUC*-OE lines was significantly higher than that of the WT. Among them, the total sugar concentration of the *VvSUC27*-OE lines was the highest (122, 109, and 110mg/g, respectively) and was 2.24–2.51-fold higher than that of the WT (49mg/g). For the *VvSUC11*- and *VvSUC12*-OE fruits, the total sugar content was increased by an average of 1.91- and 2.17-fold that of the WT.

Titratable acidity of mature fruit was also determined in this study (Figure 4D). In total, titratable acidity of *VvSUC11*-OE fruits was nearly consistent with that of WT fruits at the mature fruit stage. The final titratable acidity of fruits for the *VvSUC12*-OE-2 and 5 lines significantly decreased by 1.28- and 1.20- fold that of the WT, respectively. For *VvSUC27*-OE fruits, the titratable acidity of mature fruits for the *VvSUC27*-OE-2 and 5 lines obviously decreased of 1.33- and 1.29-fold that of the WT, respectively. The total soluble solid/titratable acidity ratio was also determined by dividing the total soluble solid by titratable acidity. For *VvSUC12*- and *VvSUC27*-OE mature fruits, the ratio of all lines was significantly improved compared with that of the WT (Figure 4E).

### Ectopic Expression of *VvSUC27* Resulted in Seedless Fruit

**Figure 5:**
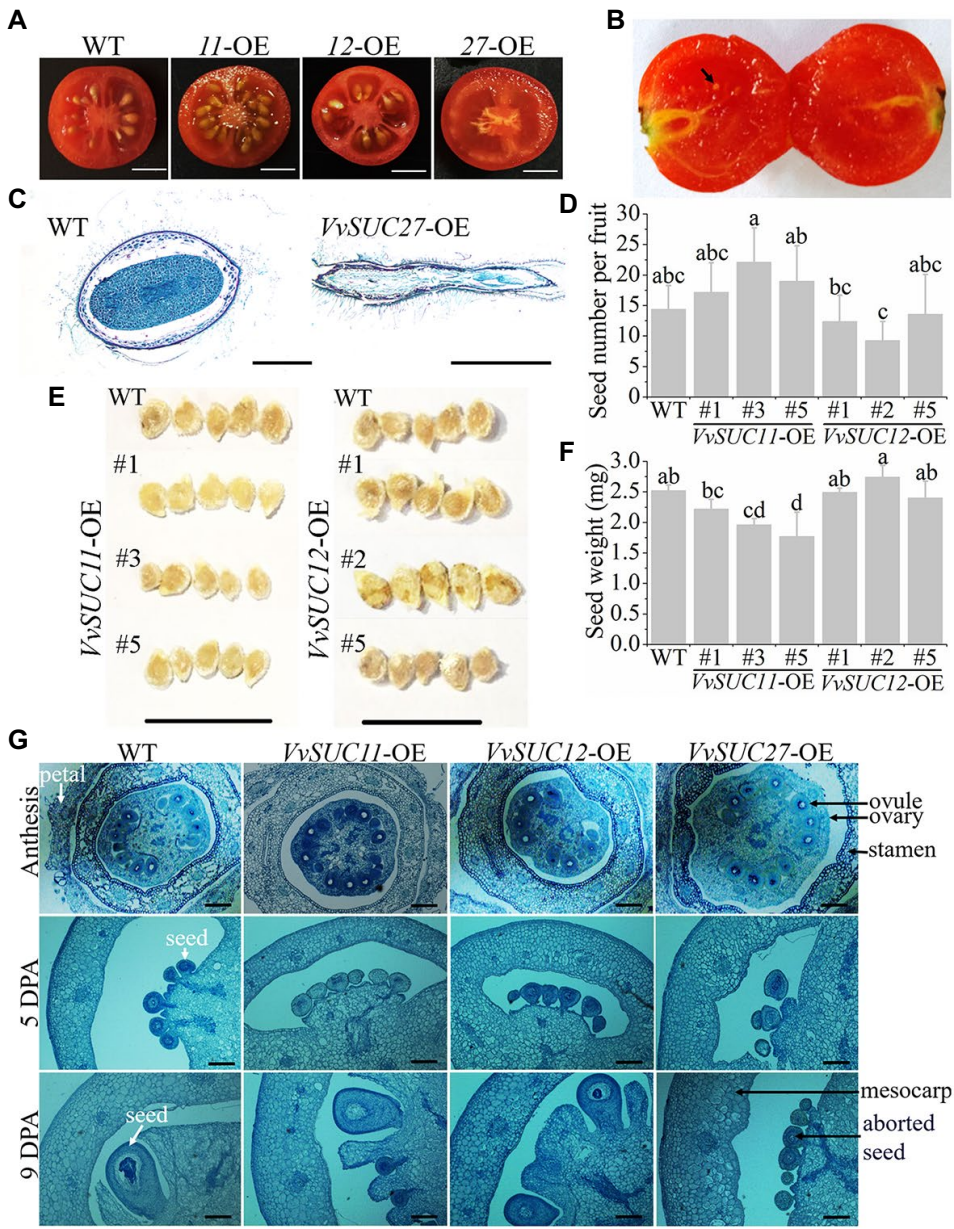
Observation of *VvSUC*-OE tomato seeds. **(A)** Crosswise cutting of fruits at the red ripe stage of the WT and *VvSUC*-OE lines. Scale bar, 50mm. **(B)** Crosswise cutting of *VvSUC27*-OE fruits at the red ripe stage. Black arrow indicates aborted seed. Scale bar, 50mm. **(C)** Normal seed from WT and aborted seed from *VvSUC27*-OE fruit observed microscopically. Cross-cut tissue slices were stained with safranin O/fast green. Scale bar, 500μm. **(D)** Comparison between the WT, *VvSUC11*-, and *VvSUC12*-OE lines regarding seed number per fruit. Error bars indicate mean±SD of twenty biological replicates. Different lowercase letters show statistically significant differences among WT, VvSUC11-, and VvSUC12-OE lines (Tukey’s test, *p*<0.05). **(E)** Comparison between the WT and *VvSUC11*- or *VvSUC12*-OE lines regarding seed shape and size. Scale bar, 1cm. **(F)** Comparison between the WT, *VvSUC11*- and *VvSUC12*-OE lines regarding seed weight. Error bars indicate mean±SD. The single seed weight was calculated from the average of the total weight of every 15 seeds, which was recorded as one experiment of ten biological replicates. Different lowercase letters show statistically significant differences among WT, VvSUC11-, and VvSUC12-OE lines (Tukey’s test, *p*<0.05). **(G)** Ovary and cell division phase of the WT and *VvSUC*-OE lines observed microscopically. Anthesis, 5 and 9days post-anthesis (DPA) of the WT and *VvSUC*-OE lines were selected to observe the ovule and seed development. Cross-cut tissue slices were stained with safranin O/fast green. Ovule, seed, aborded seed and some other parts were indicated by arrows and noted beside. Scale bar, 200μm.

Crosswise cutting of fruits at the red ripe stage of the WT and *VvSUC*-OE lines was conducted to determine that the *VvSUC27*-OE fruits were seedless, and the *VvSUC11*- and *VvSUC12*-OE seed numbers were different (Figure 5A). Crosswise cutting of *VvSUT27*-OE fruits at the red ripe stage was observed, and the aborded seeds were detected in *VvSUC27*-OE fruits (Figure 5B). The seeds from WT and *VvSUC27*-OE fruit were crosswise cut, stained with safranin O/fast green, and then observed microscopically (Figure 5C). The seed from *VvSUC27*-OE fruit was shriveled and obviously aborded compared with that of the WT.

It seemed that the *VvSUC11*-OE-3 and *VvSUC11*-OE-5 fruit produced more seeds, whereas the *VvSUC12*-OE-1 and *VvSUC12*-OE-2 fruit produced fewer seeds compared with each other; however, there was no significant difference compared with that of the WT (Figure 5D). Furthermore, five seeds from each line were set up together, and it was observed that the *VvSUC11*-OE fruit produced smaller seeds, especially those of *VvSUC11*-OE-3 and *VvSUC11*-OE-5 (Figure 5E). *VvSUC12*-OE fruit produced seeds of normal size, except for *VvSUC12*-OE-2 (Figure 5E). Seed weight was further determined: Accordingly, the weight of the *VvSUC11*-OE seeds was decreased, whereas seed weight of *VvSUC12*-OE remained unchanged compared with that of the WT (Figure 5F). The ovary and cell division phase of the WT and *VvSUC*-OE lines were cross-cut and stained with toluidine blue to visualize the formation of seeds (Figure 5G). The ovules and seeds of each *VvSUC*-OE line and the WT were similar at anthesis and 5 DPA. Furthermore, both *VvSUC11*- and *VvSUC12*-OE seeds were similar to those of the WT, whereas *VvSUC27*-OE seeds aborted at 9 DPA. The phenotypes of *VvSUC27*-OE aborted seeds at 9 DPA were similar to those at 5 DPA; therefore, we inferred that *VvSUC27*-OE seeds ceased development and stenospermocarpy at 5 DPA.

### Ectopic Expression of *VvSUC27* Impaired the Expression of Plant Hormone-Related Genes

*VvSUC27*-OE seeds stopped developing at 5 DPA, which caused the *VvSUC27*-OE lines to produce seedless fruits. Considering the developing seeds from each *VvSUC*-OE line and the WT were similar and *VvSUC27*-OE seeds ceased development at 5 DPA (Figure 5G), the entire fruits were ground up in this study. However, the results excluded the differentially expressed genes (DEGs) with seed-specific expression, because the developing seeds had not been manually removed from fruits. RNA-seq was conducted to discover the DEGs from T_1_ transgenic lines of *VvSUC11*-OE-5 and *VvSUC12*-OE-5, as well as the T_0_ transgenic line of *VvSUC27*-OE-5 fruits and the WT at 5 DPA. The RNA-seq experiment was repeated with three independent biological replicates. Raw reads were screened to obtain clean reads, and the effective rate was over 97% (Supplementary Table S2).

**Figure 6:**
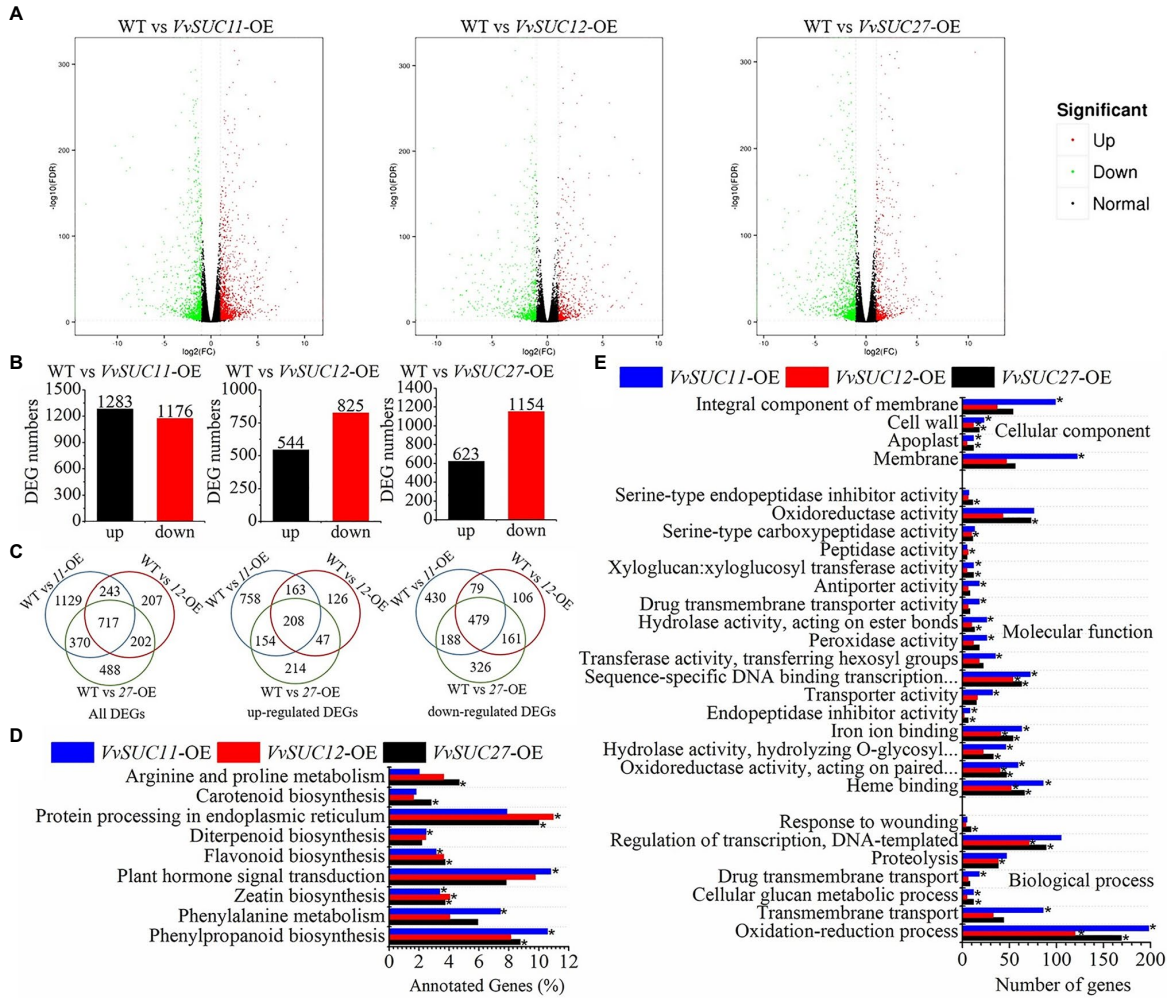
Analyses of differentially expressed genes (DEGs) in *VvSUC*-OE fruits. | log_2_^FoldChange^ |>1 and FDR<0.01 were used as thresholds to select the DEGs. **(A)** Volcano diagrams of DEGs in *VvSUCs*-OE fruits compared with WT fruits at 5 DPA. Green spots indicated DEGs whose expression was less than half that of the WT. Red spots displayed DEGs whose expression was more than double that of the WT. **(B)** Number of up- and downregulated DEGs in each *VvSUC*-OE fruit compared with that of the WT. **(C)** Venn diagrams showing all, upregulated, and downregulated DEGs that were commonly expressed in *VvSUC11*-, *VvSUC12*-, and *VvSUC27*-fruits compared with that of the WT, respectively. **(D)** KEGG pathway enrichment analysis of DEGs in 5 DPA tomato fruits of *VvSUCs*-OE lines. ^*^indicates a significantly enriched term (corrected value of *p*<0.05). **(E)** GO enrichment analysis of DEGs in 5 DPA tomato fruits of *VvSUCs*-OE lines. “Oxidoreductase activity, acting on paired…,” “Hydrolase activity, hydrolyzing O-glycosyl…,” and “Sequence-specific DNA binding transcription…” were short for “Oxidoreductase activity, acting on paired donors, with incorporation or reduction of molecular oxygen, NAD(P)H as one donor, and incorporation of two atoms of oxygen into one donor,” “Hydrolase activity, hydrolyzing O-glycosyl compounds,” and “Sequence-specific DNA binding transcription factor activity.” ^*^indicates a significantly enriched term (corrected value of *p*<0.05).

Additionally, all clean reads were mapped to the tomato reference genome, and over 80% of clean reads were mapped in each sample file, ensuring that the RNA-seq results were credible. The fragments per kilobase of transcript per million fragments mapped (FPKM) of *VvSUC11*, *VvSUC12*, and *VvSUC27* were analyzed to verify that experimental materials of *VvSUC11*-, *VvSUC12*-, and *VvSUC27*-OE fruits were the transgenic fruits (Supplementary Table S3). Compared with the WT, thousands of genes were significantly differentially expressed in *VvSUC11*-, *VvSUC12*-, and *VvSUC27*-OE fruits using the threshold for DEGs of | log_2_^FoldChange^ |>1 and a false discovery rate (FDR)<0.01 for either sample (Figure 6A; Supplementary Table S4). There were nearly equal numbers of upregulated DEGs and downregulated DEGs in the *VvSUC11*-OE fruits, whereas downregulated DEGs occurred more frequently than upregulated DEGs in the *VvSUC12*-OE and *VvSUC27*-OE fruits (Figures 6B,C). Some annotated DEGs are listed in Supplementary Table S5. These data suggest that *VvSUCs* modulate the transcription of genes during seed formation.

**Figure 7:**
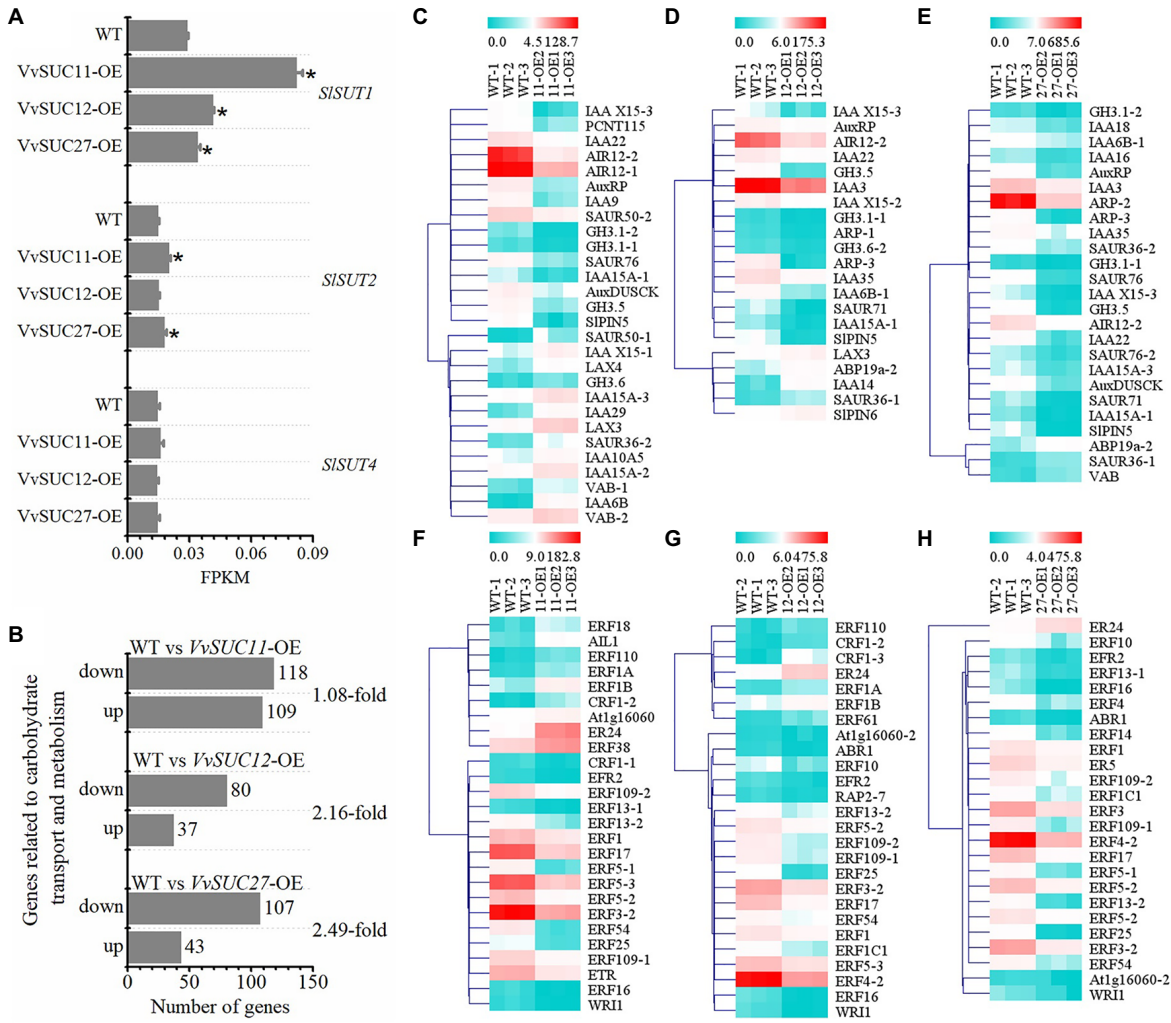
Identification of genes related to *SlSUTs*, carbohydrate transport and metabolism, auxin and ethylene biosynthesis, and signaling. log2FC is short for log_2_^FoldChange^. **(A)** Comparison between the WT and each *VvSUC*-OE line regarding FPKM of *SlSUT1*, *SlSUT2*, and *SlSUT4*. **(B)** Number of upregulated and downregulated genes related to carbohydrate transport and metabolism pathway. **(C–E)** Heat map of genes related to auxin biosynthesis and signaling between the WT and each *VvSUC*-OE fruit. IAA, SAUR, or PCNT115, auxin-induced protein or auxin-responsive protein; GH, indole-3-acetic acid-amido synthetase; AIR, auxin-induced in root culture protein; ARP, auxin-repressed 12.5kDa protein-like; AuxRP, auxin-regulated protein; SlPIN, auxin efflux facilitator; VAB, auxin canalization; ABP, auxin-binding protein; AuxDUSCK, auxin-regulated dual-specificity cytosolic kinase; LAX, auxin influx carrier (AUX1 LAX family). **(F–H)** Heat map of genes related to ethylene biosynthesis and signaling between WT and each *VvSUC*-OE fruit. Abbreviations: AIL1 or At1g16060, AP2-like ethylene-responsive transcription factor; CRF, WRI1, ABR1, RAP2, or ERF, ethylene-responsive transcription factor; ER, ethylene-responsive transcriptional coactivator; ETR, ethylene-binding protein. Statistically significant differences (*p*<0.05) are indicated by asterisks.

It has been reported that several auxin- and ethylene-associated genes are downregulated in seedless fruits (Martinelli et al., 2009). High differential expression could also occur in plant hormone signal transduction in transgenic fruit (Figures 6D,E). Genes involved in sucrose transport, carbohydrate transport, and metabolism, as well as auxin and ethylene biosynthesis and signaling, were confirmed to be differentially regulated between *VvSUCs*-OE and WT fruits (Figure 7). Compared with the WT, the functional characterization showed that the expression level (shown as FPKM) of *SlSUT1* was significantly improved in *VvSUC11*-, *VvSUC12*-, and *VvSUC27*-OE fruits. Similarly, the expression level of *SlSUT2* was increased in *VvSUC11*- and *VvSUC27*-OE fruits, whereas the expression level of *SlSUT4* was not significantly different (Figure 7A). Additionally, the DEGs involved in carbohydrate transport and metabolism were also analyzed; the downregulated genes were 1.08-, 2.16-, and 2.49-fold greater than the upregulated genes in *VvSUC11*-, *VvSUC12*-, and *VvSUC27*-OE fruits, respectively (Figure 7B) and are listed in Supplementary Table S6. Furthermore, a large proportion of auxin- and ethylene-associated genes were also downregulated in *VvSUC27*-OE seedless fruits (Figures 7C–H; selected from all annotated DEGs listed in Supplementary Table S5). For auxin biosynthesis and signaling associated DEGs, 13 upregulated and 15 downregulated genes were detected in normal seed setting in *VvSUC11*-OE fruits compared with those of the WT (Figure 7C), 5 upregulated and 16 downregulated genes were detected in *VvSUC12*-OE fruits (Figure 7D), while 22 downregulated and only three upregulated genes were found in *VvSUC27*-OE seedless fruits (Figure 7E). Genes associated with ethylene biosynthesis and signaling were also chosen for expression profiling compared with that of the WT; 9 upregulated and 17 downregulated genes were detected in *VvSUC11*-OE fruits (Figure 7F). Seven upregulated and 19 downregulated genes were detected in *VvSUC12*-OE fruits (Figure 7G), whereas most of the genes (24 genes) were downregulated, and only one gene was upregulated in the *VvSUC27*-OE seedless fruits (Figure 7H). These results suggest that *VvSUCs*, especially *VvSUC27*, inhibit auxin and ethylene production by regulating the transcription of auxin- and ethylene-related genes.

## Discussion

### Ectopic Expression of *VvSUC27* Induced Stenospermocarpy and Altered Fruit Quality

In many fruits, seeds are an undesirable feature, presenting a hard or leathery texture, bitter taste, and harmful toxic compounds. Therefore, seedlessness is a desirable trait in many commercially grown fruit crops if undesirable changes to quality can be avoided (Martinelli et al., 2009). Tomatoes are an important crop worldwide and a model for the study of fruit set and development. Therefore, harnessing seedlessness in tomatoes has been an important breeding objective (Picarella and Mazzucato, 2018). Previous reports have generally focused on describing reverse genetics experiments (Marti et al., 2007; Klap et al., 2017; Joldersma and Liu, 2018). Stenospermocarpy is the most common mechanism of parthenocarpy in seedless grape varieties, where pollination and fertilization occur but the embryo subsequently aborts, leaving undeveloped seeds or seed traces (Mejia et al., 2011). This work is the first to demonstrate the ability of VvSUC27 to induce seedless fruit (stenospermocarpy; only a few pollen grains could germinate) and improve fruit quality. As a strong sink organ, flowers consist of male and female floral organs, including the petals, style, and pollen. The development of floral organs is dependent on sucrose unloading from the phloem, which requires the participation of SUT. In this context, among the three VvSUCs, VvSUC27 had the most influential role in this process. Several studies have reported that SUTs play a significant role in pollen and seed development. Tomato plants transformed with a SlSUT2 antisense construct were exclusively affected by impairing pollen tube growth, preventing pollination, and causing seedless fruits (Hackel et al., 2006). AtSUC1 insertional mutants produce defective pollen (Sivitz et al., 2008). Additionally, RNAi-mediated downregulation of *CsSUT1* expression induced male sterility in cucumbers (Sun et al., 2019). However, similar to the suppression of other plant *SUTs*, ectopic expression of *VvSUC27* resulted in significantly enlarged petals and pistils, abnormal stigma, and much less abundant and shrunken pollen, whereas ectopic expression of *VvSUC11* and *VvSUC12* produced flowers similar to those of the WT (Figures 1, 2). Thus, it could be inferred that one reason for stenospermocarpy is the ectopic expression of *VvSUC27*, which leads to a consequential reduction in nutrient delivery to the pollen and stigma.

Seedless fruits are more desirable because of their lower acidity and higher proportion of soluble solids compared with seeded fruits (Sell et al., 1953; Martinelli et al., 2009). Seedless tomatoes with good taste would be preferred among fresh market fruits. Ectopic expression of *VvSUC27* not only induced tomato fruit stenospermocarpy, but also significantly increased fruit width, height, and firmness compared with those of the WT (Figure 3). It has been reported that overexpression of *MdSUT2.2* could result in the accumulation of more total soluble sugars and sucrose in apples (Ma et al., 2017, 2019a), whereas ectopic expression of *PbSUT2* led to an improvement in the content of sucrose while decreasing the contents of glucose, fructose, and total soluble sugars in mature tomato fruits. Ripe fruits of commercial tomato variants have equimolar concentrations of glucose and fructose, but they contain little sucrose (Klee and Giovannoni, 2011). Here, ectopic expression of all three *VvSUCs* improved soluble sugar contents and the concentration of glucose and fructose in mature tomato fruits, especially ectopic expression of *VvSUC27* (Figure 4). For *VvSUC12*- and *VvSUC27*-OE mature fruits, the titratable acidity decreased and total soluble solid/titratable acidity ratio of all lines was significantly improved compared with that of the WT (Figure 4E). Furthermore, compared with the WT, two of the *VvSUC11*-OE lines and all of the *VvSUC12*-OE lines significantly improved the sucrose content of mature fruit, while no sucrose could be detected in *VvSUC27*-OE lines (Figure 4C). The different sucrose contents in the mature fruit may be due to the different effects of *VvSUCs* on cell wall bound and vacuolar invertases on fruit sink strength (Yelle et al., 1991; Klann et al., 1996; Jin et al., 2009; Zanor et al., 2009). Thus, it can be inferred that all three *VvSUCs* participated in the consequential sugar accumulation in the sink. The positive traits of improved firmness, increased sugar content, and stenospermocarpy on *VvSUC27*-OE fruits would be desirable in fresh market fruits.

### The Downregulated Genes in Carbohydrate Transport and Metabolism, and Auxin- and Ethylene-Related Signaling Pathways May Underlie Stenospermocarpy

There could be a very complex mechanism underlying seedless crops, with many genes being reported as responsible for this phenomenon (Marti et al., 2007; Klap et al., 2017; Cong et al., 2019; Wen et al., 2019; Wang et al., 2020), along with alterations in hormone levels and carbohydrate supply. Many studies have shown that genes involved in carbohydrate transport and metabolism are related to seedlessness. Attributed to pollination failure and impaired male and female fertilities, significant reductions in viable seeds were observed after silencing vacuolar invertase genes in cotton, in addition to a reduction in the expression of genes involved in starch metabolism (Wang and Ruan, 2016). Downregulation of *CsSUT1* caused a significant downregulation in the expression of many genes related to carbohydrate metabolism and sugar transport in male flowers, which is one of the main causes of male sterility (Sun et al., 2019). Therefore, direct changes in seedless fruits induced by genetic transformation have been well characterized at anthesis. In this study, five DPA fruits were chosen to emphasize the differences between transgenic and WT fruits during seed formation. The expression of most genes related to carbohydrate metabolism and sugar transport was downregulated in *VvSUC12*- and especially in *VvSUC27*-OE lines, whereas nearly equal numbers of genes were downregulated and upregulated in more seeded *VvSUC11*-OE lines (Figure 7B). These results suggest that ectopic expression of *VvSUC27* could downregulate the expression of genes involved in carbohydrate metabolism and sugar transport during early fruit development, which may regulate the growth and development of seeds.

Seedless fruits are often reported to have a longer shelf life than seeded fruits as seeds can produce hormones, such as ethylene, which triggers senescence (Fei et al., 2004). Previously published data showed that the downregulation of IAA-responsive genes or Aux/IAA family members resulted in seedless fruits (Goetz et al., 2007; de Jong et al., 2009; Yang et al., 2013). For SUT, the knockdown of *CsSUT1* in cucumbers at the twelfth stage of male flowers caused changes in genes associated with auxin signaling (Sun et al., 2019). In our study, only a small proportion of genes involved in auxin- and ethylene-related signaling pathways were upregulated in all transgenic fruits compared with the WT (Figure 7). The more seeds the transgenic tomato had, the lower the proportion of downregulated genes; therefore, the proportion of downregulated genes in more seeded *VvSUC11*-OE fruits was lower than that in *VvSUC12*-OE fruits, especially seedless *VvSUC27*-OE fruits. In this regard, we demonstrated that compared with those of the WT, the expression of most genes related to auxin-related signaling pathways (*IAA*, *SAUR*, *PCNT115*, *GH*, *AIR*, *ARP*, *AuxRP*, *AuxRP*, *SlPIN*, *VAB*, *ABP*, *AuxDUSCK*, and *AUX1 LAX* family) was all downregulated in *VvSUC27*-OE fruits at 5 DPA. However, three genes, namely, *ABP19a-2*, *SAUR36-1*, and *VAB*, were not. Concerning the expression of genes related to the ethylene-related signaling pathway, (AP2-like) ethylene-responsive transcription factor (*AIL1* and *At1g16060*), ethylene-responsive transcription factor (*CRF*, *WRI1*, *ABR1*, *RAP2*, and *ERF*), ethylene-responsive transcriptional coactivator (*ER*), and ethylene-binding protein were all downregulated, except for one gene, *ER24*. Our results were similar to the report that found several ethylene- and IAA-associated genes were downregulated in transgenic seedless tomatoes at the breaker stage (Martinelli et al., 2009). These results suggest that VvSUC27 could downregulate the expression of genes involved in auxin- and ethylene-related signaling pathways during early fruit development, which may regulate the growth and development of seeds. The genes which were specifically expressed in seeds were not included since the developing seeds were unremoved from fruits.

In summary, ectopic expression of *VvSUC27* in tomatoes caused a consequential reduction in nutrient delivery to the pollen, with a subsequent downregulation of the genes involved in carbohydrate metabolism and sugar transport, along with auxin- and ethylene-related signaling pathways during early fruit development, which may regulate the growth and development of seeds. This work is the first to demonstrate the ability of *VvSUC27* to induce seedless and high-quality fruits, which would be an effective and commercially desirable approach for bioengineering stenospermocarpy in tomatoes and other crops.

## Data Availability Statement

The datasets presented in this study can be found in online repositories. The names of the repository/repositories and accession number(s) can be found in the article/Supplementary Material.

## Author Contributions

YZ led the project. YC, WT, ZD, JY, WD, HG, JX, NZ, and JW performed the experiments. YZ, YC, LY, LZ, and QM designed and supervised the experiments. YZ and YC wrote and edited the paper. All authors contributed to the article and approved the submitted version.

## Funding

This research was supported by a grant from the National Natural Science Foundation of China (Grant No. 30900969), Bagui Young Scholars’ Special Fund of Guangxi and the Special Fund for the Central Government Guides Local Science and Technology Development (Guike ZY21195039), China Natural Science Foundation of Heilongjiang Province (Grant No. QC2017024), and the Project for Extramural Scientists of State Key Laboratory of Agrobiotechnology (2020SKLAB6-8).

## Conflict of Interest

The authors declare that the research was conducted in the absence of any commercial or financial relationships that could be construed as a potential conflict of interest.

## Publisher’s Note

All claims expressed in this article are solely those of the authors and do not necessarily represent those of their affiliated organizations, or those of the publisher, the editors and the reviewers. Any product that may be evaluated in this article, or claim that may be made by its manufacturer, is not guaranteed or endorsed by the publisher.

## Footnotes

^1^ ftp://ftp.ncbi.nlm.nih.gov/genomes/all/GCF/000/188/115/GCF_000188115.3_SL2.50/GCF_000188115.3_SL2.50_genomic.fna.gz

^2^ https://www.ncbi.nlm.nih.gov/geo/query/acc.cgi?acc=GSE161505
