# Supplementary material for: Ectopic Expression of VvSUC27 Induces Stenospermocarpy and Sugar Accumulation in Tomato Fruits
Source: Front Plant Sci. 2021 Nov 17;12:759047. doi: 10.3389/fpls.2021.759047 (PMC8637806; doi:10.3389/fpls.2021.759047)
Supplement: Supplementary file 1 [file Data_Sheet_1.DOCX]

## Supplementary Figures


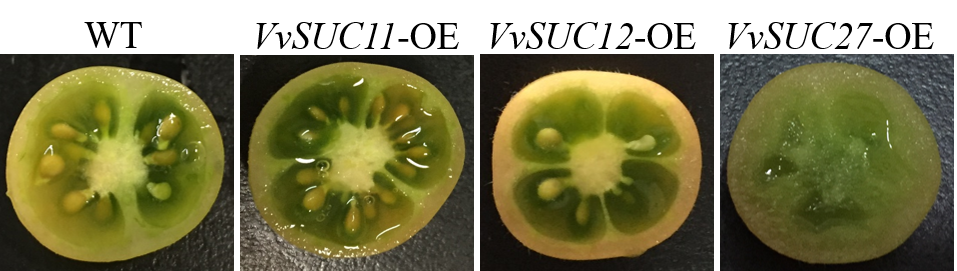


**Supplementary Figure 1.** Comparison between WT and each *VvSUC*-OE on the phenotype of T_0_ cross-cut tomato fruits at break period.


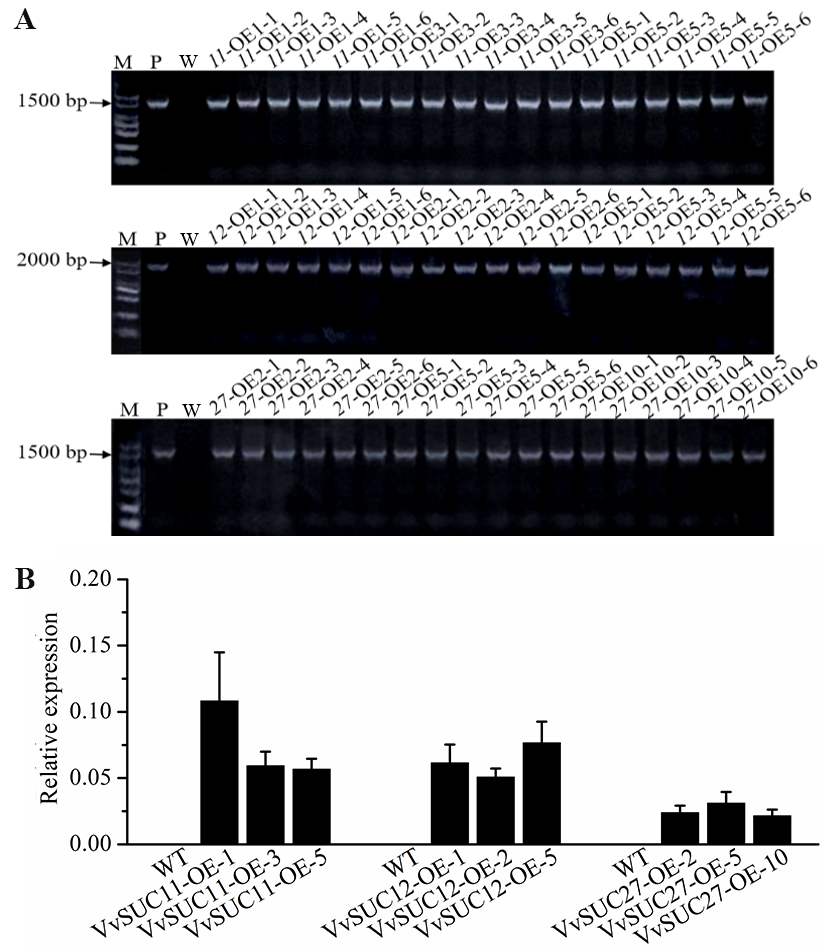


**Supplementary Figure 2.** PCR **(**A**)** and qRT-PCR (B) verification of *VvSUC* transformants. Gene transcript levels were normalized against three reference gene (*SlEF-1α*, *SlGADPH*, and *SlActin*). Data are expressed as the mean ± S.D. from three biological replicates.


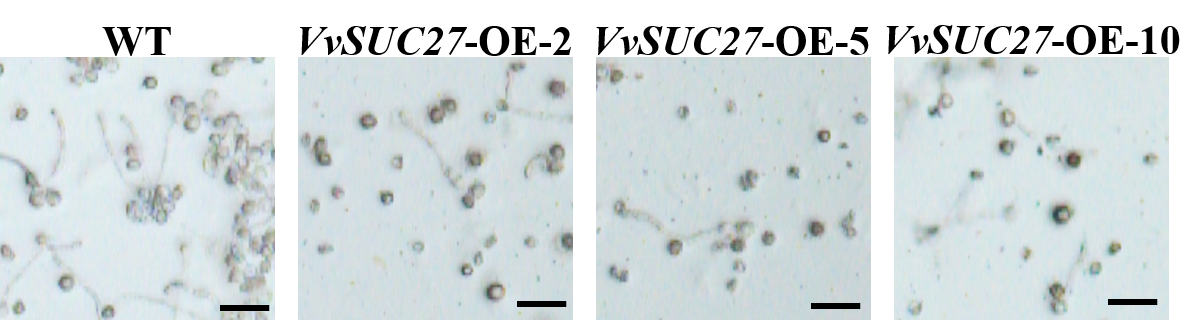


**Supplementary Figure 3.** Pollen germination and growth *in vitro*. Scale bar, 100 μm.

## Supplementary Tables

**Supplementary Table 1.** Primers used in this study.

| **Gene name** | **Primer Sequence (5’-3’)** |
| --- | --- |
| attB1,2-*VvSUC11* | For：AAAAAGCAGGCTAGATGGCGGTCCCTGGGG  Rev：AGAAAGCTGGGTCTCATGTGTGGACCCTGGATTT |
| attB1,2-*VvSUC12* | For：AAAAAGCAGGCTAGATGCCGGAGACGATGGACG  Rev：AGAAAGCTGGGTCTCACCTCATTGAGGCAGGGA |
| attB1,2-*VvSUC27* | For：AAAAAGCAGGCTAGATGGAGTTAGCCAAGCCTTCTTC  Rev：AGAAAGCTGGGTCTTAAGACGACGGCTGAGTCCTC |
| attB-adapter | For：GGGGACAAGTTTGTACAAAAAAGCAGGCT  Rev：GGGGACCACTTTGTACAAGAAAGCTGGGT |
| *SlEF-1a*  (qRT-PCR) | For：TACTGGTGGTTTTGAAGCTG  Rev：ACTTCCTTCACGATTTCATCATA |
| *SlGAPDH*  (qRT-PCR) | For：GGTGCCAAGAAGGTTGTGAT  Rev：TTTTCTGGGTGGCAGTCAT |
| *SlActin*  (qRT-PCR) | For：CAGCAGATGTGGATCTCAAA  Rev：CTGTGGACAATGGAAGGAC |

**Supplementary Table 2.** Analysis the data of RNA-seq

| **Sample Name** | **Raw Reads** | **Clean Reads** | **Raw Bases**  **(Gb)** | **Clean Bases**  **(Gb)** | **Effective Rate**  **(%)** | **Uniq Mapped Reads** | **Q30 content**  **(%)** |
| --- | --- | --- | --- | --- | --- | --- | --- |
| *VvSUC11*-OE1 | 50332156 | 49107902 | 7.55 | 7.37 | 97.57 | 43826309 (89.24%) | 94.67 |
| *VvSUC11*-OE2 | 49991240 | 48787752 | 7.50 | 7.32 | 97.59 | 42159273 (86.41%) | 94.72 |
| *VvSUC11*-OE3 | 50018376 | 48721474 | 7.50 | 7.31 | 97.41 | 40130902 (82.37%) | 94.06 |
| *VvSUC12*-OE1 | 50125142 | 49037136 | 7.52 | 7.36 | 97.83 | 43444013 (88.59%) | 94.44 |
| *VvSUC12*-OE2 | 50157210 | 48883934 | 7.52 | 7.33 | 97.46 | 43075693 (88.12%) | 92.31 |
| *VvSUC12*-OE3 | 50172544 | 48752736 | 7.53 | 7.31 | 97.17 | 42668205 (87.52%) | 92.10 |
| *VvSUC27*-OE1 | 50103050 | 49150896 | 7.52 | 7.37 | 98.10 | 44949656 (91.45%) | 95.17 |
| *VvSUC27*-OE2 | 50206248 | 49100488 | 7.53 | 7.37 | 97.80 | 43006330 (87.59%) | 94.93 |
| *VvSUC27*-OE3 | 49982618 | 49047136 | 7.50 | 7.36 | 98.13 | 44088934 (89.89%) | 94.41 |
| WT-1 | 41148970 | 40276852 | 6.17 | 6.04 | 97.88 | 36369147 (90.30%) | 94.96 |
| WT-2 | 43834364 | 42744448 | 6.58 | 6.41 | 97.51 | 38924813 (91.06%) | 95.30 |
| WT-3 | 50170522 | 49169906 | 7.53 | 7.38 | 98.01 | 44938028 (91.39%) | 95.20 |

**Supplementary Table 3.** FPKM of *VvSUCs* in *VvSUC11*-, *VvSUC12*-, and *VvSUC27*-OE fruits, respectively.

| **Sample Name** | ***VvSUC11*-OE** | ***VvSUC12*-OE** | ***VvSUC27*-OE** |
| --- | --- | --- | --- |
| *VvSUC11*-OE1 | 0.023869 | 0 | 0 |
| *VvSUC11*-OE2 | 0.024779 | 0 | 0 |
| *VvSUC11*-OE3 | 0.024815 | 0 | 0 |
| *VvSUC12*-OE1 | 0 | 0.003554 | 0 |
| *VvSUC12*-OE2 | 0 | 0.002856 | 0 |
| *VvSUC12*-OE3 | 0 | 0.002604 | 0 |
| *VvSUC27*-OE1 | 0 | 0 | 0.032795 |
| *VvSUC27*-OE2 | 0 | 0 | 0.031713 |
| *VvSUC27*-OE3 | 0 | 0 | 0.032021 |
| WT-1 | 0 | 0 | 0 |
| WT-2 | 0 | 0 | 0 |
| WT-3 | 0 | 0 | 0 |

Note: FPKM standardization were conducted by coffnorm.
